# Dipole charge density mapping integrated in remote magnetic navigation: First-in-human feasibility study

**DOI:** 10.1016/j.ijcha.2022.101095

**Published:** 2022-07-21

**Authors:** Rita B. Gagyi, Anna M.E. Noten, Sip Wijchers, Sing-Chien Yap, Rohit E. Bhagwandien, Mark G. Hoogendijk, Tamas Szili-Torok

**Affiliations:** Erasmus Medical Center, Rotterdam, the Netherlands

**Keywords:** Atrial arrhythmia, Catheter ablation, Robotic navigation, Mapping and imaging

## Abstract

**Aims:**

Robotic magnetic navigation (RMN) provides increased catheter precision and stability. Formerly, only the CARTO 3 mapping system was integrated with the RMN system (CARTO-RMN). Recently, a novel high-resolution non-contact mapping system (AcQMap) has been integrated with the RMN system (AcQMap-RMN) for the treatment of atrial fibrillation (AF) and atrial tachycardias (AT). We aim to compare the safety, efficiency, and efficacy of AcQMap-RMN with CARTO-RMN guided catheter ablation (CA) procedures.

**Material and methods:**

In this prospective registry, procedural safety efficiency and outcome data from total of 238 consecutive patients (147 AcQMap-RMN and 91 CARTO-RMN patients) were compared.

**Results:**

AcQMap-RMN is non-inferior in the primary endpoint of safety as compared to CARTO-RMN across the whole group (overall procedural complications in 5 (3.4%) vs. 3 (3.3%) patients, p = 1.0). Overall procedure durations were longer and associated with more fluoroscopy use with AcQMap-RMN (172.5 vs. 129.6 min, p < 0.01; 181.0 vs. 131.0 mGy, p = 0.02, respectively). Procedure duration and fluoroscopy use decreased significantly between the first 30 and the last 30 AcQMap-RMN procedures. The AcQMap-RMN system had fewer recurrences after persistent AF ablations and was non-inferior in paroxysmal AF patients compared to CARTO-RMN at 12 months (36.6% vs. 75.0%, p = 0.04, PAF 6.6% vs. 12.5%, p = 0.58; respectively). CA of AT outcomes were better using the AcQMap-RMN system (1 year recurrence 17.1% vs. 38.7%, p < 0.05).

**Conclusion:**

AcQMap-RMN integration has no negative impact on the excellent safety profile of RMN guided ablations. It improves outcomes of CA procedures for persAF and AT but requires longer procedure times and higher fluoroscopy use during the initial learning phase.

## 1 Introduction

Catheter ablation (CA) has become a well-established first-line therapy for a broad spectrum of arrhythmias [1], [2], [3]. Robotic magnetic navigation (RMN) has previously been utilized in CA for various types of arrhythmias [4], [5], [6], [7]. RMN allows for high-precision computer-guided catheter movements, and its atraumatic catheter design has a superior safety profile without compromising the efficiency of CA [6], [8], [9], [10], [11].

Until recently, only the CARTO 3 (Version 6) (Biosense-Webster Inc., Diamond Bar, CA, USA) mapping system was fully integrated with the RMN system (Niobe ES, Stereotaxis, St. Louis, MO, USA) allowing mapping of arrhythmias in the atria and ventricles (CARTO-RMN). Recently, a novel high-resolution noncontact mapping system (AcQMap, Acutus Medical, Carlsbad, CA, USA) for mapping atrial arrhythmias was introduced, which is fully integrated with the RMN system (AcQMap-RMN).

The aim of this first-in-human experience study was to compare safety, efficiency, and efficacy of AcQMap-RMN integration in CA ablation procedures with CARTO-RMN mapping for patients with atrial fibrillation (AF) and atrial tachycardias (ATs).

## 2 Material and methods

### 2.1 Primary hypothesis and study design

This study is based on a prospective registry. The institutional medical ethics committee approved the data collection for this study and concluded that it did not fall under the Medical Research Involving Human Subjects Act (SERCA-2, MEC-2021-0299).

The primary hypothesis of this comparative non-inferiority study was that the AcQMap-RMN integration is safe and feasible for AF and AT ablation. Furthermore, we hypothesized that it offers improvements in efficiency and efficacy compared to the CARTO-RMN guided ablations. The primary endpoint of this study was safety characterized by intra- and post-procedural complications. The secondary endpoints were procedural efficiency and efficacy characterized by procedure time, ablation time, radiation time, radiation dose and success rate.

We analyzed data of all consecutive patients undergoing mapping and CA using the AcQMap-RMN system. Based on the power calculation, the study population was compared to an age and sex matched cohort of control patients mapped with the CARTO-RMN system. Procedures from both the study and control patient population were performed during the same inclusion period (between March of 2018 and December of 2021), by the same group of senior electrophysiologists, and there were no differences regarding any demographic data. The inclusion criteria were documented AF or AT on ECG, Holter monitoring, or previous CA procedure with documented recurrences.

### 2.2 Definitions

We defined de novo procedures as cases when no previous invasive arrhythmia treatments were performed, and redo procedures as cases when repeat procedures were performed in patients with arrhythmia recurrences after initial ablation procedures. Major complications were defined as any procedure-related adverse event, which were life threatening, required significant surgical intervention and prolonged hospital stay or resulted in death. Minor complications were defined as procedure-related adverse events, which resulted in minimal transient impairment of a body function or damage to a body structure, or which did not require any intervention or further therapy. Puncture site complications included bleeding, hematoma formation and arteriovenous fistula formation, which required surgical intervention, prolonged hospitalization and/or a Hb drop of >1.8 mmol/L. Total procedure time was defined as the time passed from first venous puncture until the removal of sheaths. Acute success was defined as arrhythmia source elimination and/or arrhythmia termination. In patients with AF, recurrence was defined as arrhythmia lasting >30 s recorded on 12-lead ECG, or 24-hour to 7-day continuous Holter monitoring. For patients with AT any documented ATs were considered as recurrences regardless of their duration.

### 2.3 Data collection

Baseline demographic, clinical characteristics and procedural data from patients were collected from our prospective database using electronic health records (HiX version 6.1) and analyzed in accordance with the hospital institutional review board policies. The following demographic and procedural data were collected: age, sex, height, weight, BMI, date of procedure, procedure duration time, number of applications, application duration, fluoroscopy dose, AF termination, rhythm at the end of procedure, acute intra-procedural and post-procedural adverse events. Further, we collected and analyzed clinical data, such as left atrial dimension, left ventricular ejection fraction, comorbidities, and antiarrhythmic medication.

### 2.4 AcQMap and AF/AT mapping

**Fig. 1:**
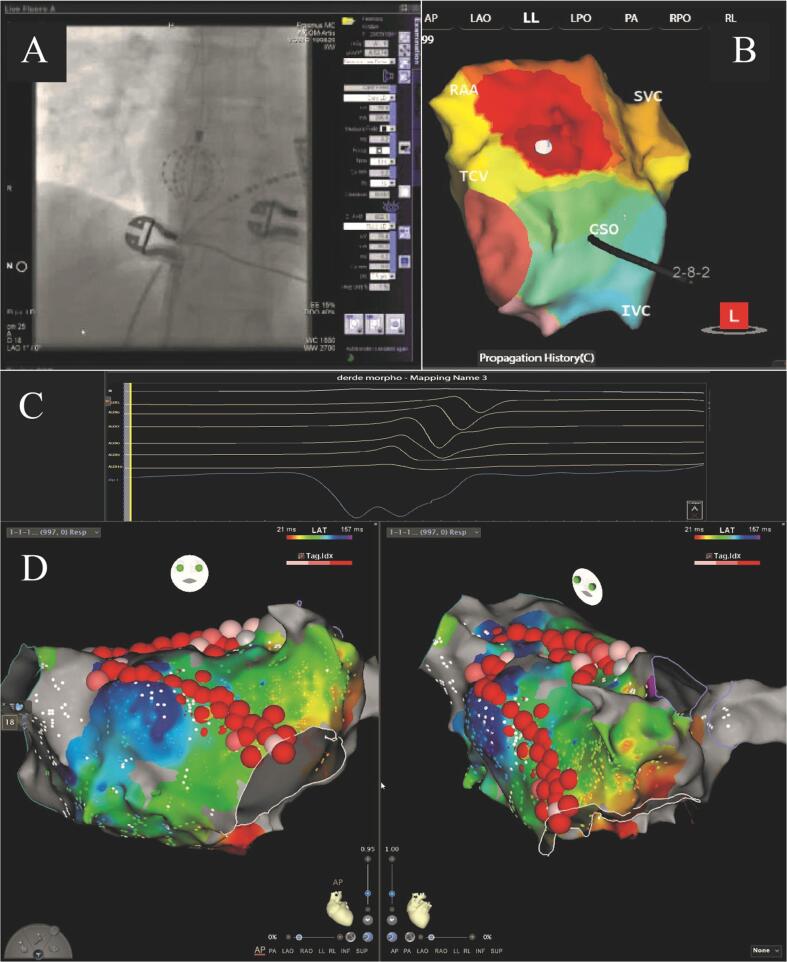
AcQMap-RMN and CARTO-RMN procedures. Panel A shows the basket catheter under fluoroscopy supervision during AcQMap mapping. Panel B shows isochronal propagation history map identifying a perinodal activation. Panel C shows unipolar charge signal. Panel D shows CARTO maps performed during post-PVI AT ablation.

The AcQMap system is a noncontact high-resolution charge density-based mapping technology that allows visualization of global atrial activation. It combines highly accurate ultrasound-based 3D endocardial anatomy reconstructions with high-resolution propagation history maps of electrical activation. The 48-pole noncontact mapping catheter (AcQMap catheter, Acutus Medical, Carlsbad, CA) has six splines, each spline incorporating eight biopotential electrodes and eight ultrasound transducers. Ultrasound is used for 3D endocardial chamber surface reconstruction, which corresponds to the end-diastolic size and shape of the atrium. Unipolar intracardiac potentials are sensed from the biopotential electrodes of the basket catheter and are processed by an inverse solution to derive the chamber-wide distribution of charge sources at the endocardial surface. The waves of activation are displayed across the 3D anatomy reconstruction through time as high-resolution propagation history maps (see Fig. 1). The noncontact nature of the AcQMap system allows for two modalities of mapping: single position and SuperMap. Single position mapping is used for unstable irregular arrhythmias such as atrial fibrillation revealing complex localized activation patterns such as focal firing, localized partial rotational activation and localized irregular activation, while SuperMap enables mapping of both non-sustained and sustained repetitive atrial rhythm. After hovering the AcQMap catheter around the chamber of interest, multiple noncontact catheter positions are time aligned based on CS activation. Beat groups are determined by morphology and separate high-resolution maps of each morphology are calculated and visualized on the anatomical shell [12].

### 2.5 AcQMap-RMN AF ablation

The noncontact AcQMap mapping catheter was introduced via a 15F (outer diameter) deflectable sheath (AcQGuide sheath, Acutus Medical, Carlsbad, CA) following transseptal access. After left atrial endocardial anatomical surface reconstruction with the AcQMap noncontact mapping system, first isolation of the pulmonary veins (PVs) was performed for de novo patients and re-isolation of the PVs was performed when needed for redo patients. CA was performed using the MagnoFlush (Medfact, Germany) ablation catheter introduced via an Agilis NxT Fr 8.5 medium curve sheath. The following settings were used: 45–50 W (posterior wall-anterior wall, respectively), 17 mL/min flow rate, maximum temperature 43℃. Patients with isolated PVs and persisting arrhythmia underwent targeted ablation of substrate guided by charge density mapping with interpreted propagation history. We administered intravenous heparin for anticoagulation with a targeted activated clotting time (ACT) > 350 s. When patients converted to atrial tachycardia/flutter during ablation, new single position maps or SuperMaps (see above) were acquired followed by targeted ablation. Conversion to sinus rhythm was achieved with electrical cardioversion (ECV) at the end of the procedures when AF persisted after CA.

### 2.6 CARTO-RMN AF ablation

After a decapolar diagnostic catheter was positioned in the coronary sinus, and a double septal puncture was performed, a Lasso catheter was advanced into the left atrium. The 4-mm tip Navistar RMT ThermoCool ablation catheter (Biosense Webster, US) was advanced into the LA via an Agilis NxT Fr 8.5 medium curve sheath and was guided by the RMN system. Atrial mapping was performed by sweeping the Lasso catheter around the LA, reconnection of PVs was checked in redo procedures. After completion of mapping, PVI or redo-PVI was performed with the following settings: maximum radiofrequency energy application 30–50 W, with temperature limit 43 °C; and 17–30 mL/min irrigation. Substrate ablation could be performed in patients with persistent atrial fibrillation at the discretion of the operator. If substrate ablation was performed, typically a posterior box lesion set was applied in accordance with our local procedural protocol. We administered intravenous heparin for anticoagulation with a targeted ACT between 270 and 300 s. ECV was performed when indicated.

### 2.7 AT ablation

An initial standard diagnostic EP study was performed for every patient diagnosed with AT. In cases when CARTO was used, we made electro-anatomic reconstructions and analyzed activation maps (see Fig. 1). When AcQMap was used, after reconstructing the endocardial anatomical surface, we overlaid high resolution charge density base maps of electrical activation using the AcQMap mapping system. Ablation was performed using the previously mentioned ablation catheters (MagnoFlush for AcQMap, and NaviStar RMT for CARTO). Intravenous heparin was administered for anticoagulation, guided by activated clotting time (>350 sec for LA and > 300 sec for RA during AcQMap-RMN procedures, and between 275 and 300 sec during CARTO-RMN procedures). We interpreted propagation history maps, identified atrial activation patterns, and performed targeted ablation.

### 2.8 AcQMap and CARTO RMN integration

The Niobe ES RMN system is a medical platform technology manufactured by Stereotaxis, Inc. (St. Louis, MO, USA). It allows remote-controlled navigation during interventional procedures. The Stereotaxis system consists of two large neodymium-iron-boron magnets positioned on either side of the patient, and it is fully integrated with fluoroscopy and electroanatomic mapping. As the system magnets move, the orientation of the magnetic field changes. The magnets on the ablation catheter move to align with this field, allowing the operator to navigate through the cardiac anatomy. This technique has been previously described [11], [13]. The new e-Contact module provides accurate real-time information on catheter-tissue contact, leading to efficient RMN-guided CA procedures [14]. Both described mapping systems are fully integrated. This allows the operator and mapper to use the RMN interface to control all steps of the procedures via the Odyssey screen.

### 2.9 Follow-up

After each procedure, patients were monitored by 24-hour telemetry. Before hospital discharge, regular access site checks, post-procedural echocardiography and ECG recordings were performed in order to screen post-procedural complications.

Based on institutional standard protocols, different follow-up routines were applied for AF and AT patients. For patients undergoing AF ablation follow-up visits were scheduled at the outpatient clinic of our department at 3, 6 and 12 months after the procedure. During the follow-up visits 24-h (at 3 and 6 months) and 7-day Holter recordings (at 12 months) were analyzed for documentation of recurrent arrhythmias. For patients with AT as the primary indication for CA, a follow-up visit at 3 months after the procedure was planned. In the cases where there was no recurrence at the follow-up visit, the patients were discharged and were considered as successful throughout the entire follow-up period if no further recurrences reported during the 12 months post-ablation period. However, post-PVI/post-MAZE AT patients were followed up based on the PVI follow-up protocol with a routine 6 and 12 months hospital visit. Patients with recurrences were considered for repeated CA procedure or were identified as ablation failures. Because of the follow up differences between the two arrhythmia groups, in this manuscript we report on 12-months outcomes for AF, while we report 3-months and 12-months outcomes for AT patients.

### 2.10 Sample size calculation and statistical analysis

We used a non-inferiority approach for the sample size calculation with the intention to avoid lack of power. Sample size was calculated based on the hypothesis that the expected AcQMap-RMN integration safety corresponds to the CARTO-RMN integration safety in atrial arrhythmia ablation. Using a non-inferiority margin of 5% for the procedural-related complication rate, the total sample size to provide statistical power of 0.8, type I error of 0.2, and case to control ratio 2:1, was calculated to be 243 patients (sample size treat n = 162, control n = 81). However, given that the AcQMap-RMN integration is a relatively novel approach, our local database contained a total of 147 patients. Due to the nature of the AcQMap system there was a higher number of patients referred for persistent atrial AF, therefore we included addition patient with persistent AF in the CARTO-RMN group for a better comparison. Subsequently, we calculated a power (1-beta) of 0.8 for our final sample size.

All analyses were carried out using SPSS 25.0 software. Mean and standard deviation (SD) were calculated for normally distributed continuous variables. Median and interquartile range (IQR) were computed for continuous variables with non-normal distribution. Normality of distribution was assessed with skewness. Descriptive statistics for categorical data were expressed in absolute numbers and percentages. Statistical significance was defined as p < 0.05 (two-tailed). Data showing normal distribution were compared using independent samples *t*-test, while non-normally distributed variables were analyzed using the Mann-Whitney *U* test. The chi-square test was used to compare categorical variables between groups. A learning curve was constructed as a classical chart and box plot using unpaired *t* test and Mann-Whitney *U* test to present and compare mean procedure duration and fluoroscopy use between early and late AcQMap-RMN procedures. We divided the AcQMap-RMN patients into groups of 30 and compared means of the first 30 patients to each group. Finally, we compared mean procedure duration and fluoroscopy use between late AcQMap-RMN procedures to the overall CARTO-RMN patient group. Our center has long experience with CARTO-RMN ablation procedures as standard approach. Therefore, it is highly unlikely that any significant learning curve impact is present in this patient group.

## 3 Results

### 3.1 Demographic and baseline clinical data

**Fig. 2:**
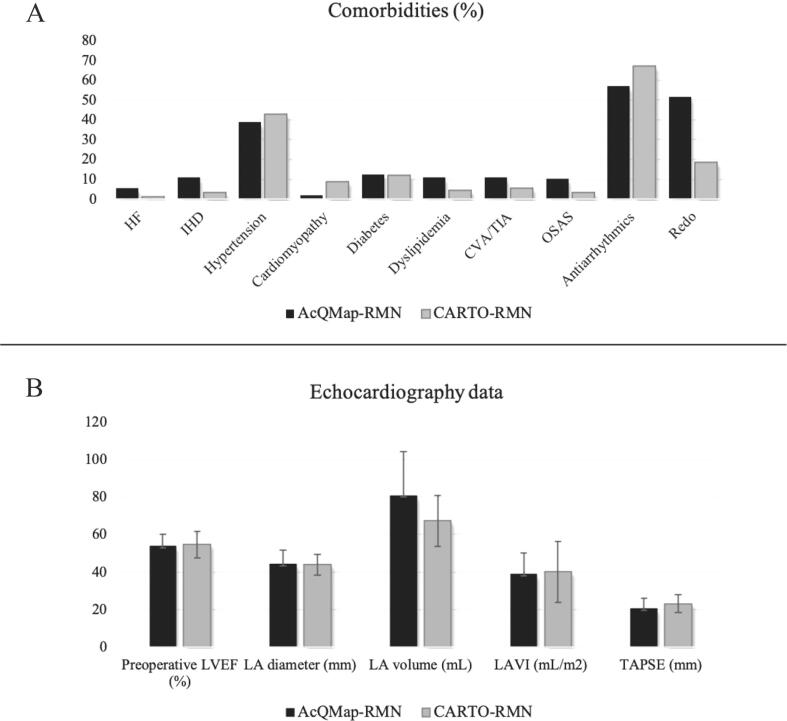
Patient demographics. Panel A describes comorbidities in our patient population for AcQMap-RMN and CARTO-RMN groups separately. The following comorbidities are listed: heart failure (HF), ischemic heart disease (IHD), hypertension, cardiomyopathy, diabetes, dyslipidemia, cerebrovascular accident or transient ischemic attack (CVA/TIA), obstructive sleep apnea syndrome (OSAS), antiarrhythmic medication. Percentage of redo procedure is also indicated. Panel B shows average echocardiography data compared between patient groups.

A total number of 147 consecutive patients treated with AcQMap-RMN, and a total number of 91 patients treated with CARTO-RMN were included in this study. Seventy-one patients had AF (male n = 47, female n = 24) in the AcQMap-RMN group, while 52 (male n = 35, female n = 17) had AF in the CARTO-RMN control group. Twenty-four patients (33.8%) had paroxysmal (PAF), 47 patients (66.2%) had persistent AF (persAF) in the AcQMap-RMN patient group; 32 patients (61.5%) had PAF and 20 (38.5%) had persAF in the control group. A total number of 76 patients were referred for CA with atrial arrhythmias, including 3 patients with inappropriate sinus tachycardia and 73 patients with AT. Out of 73 patients, 8 patients had perinodal AT. From the remaining 65 patients, 38 had de novo (58.5%), 22 had post-PVI (33.8%) and 5 had post-MAZE AT (7.7%). Focal mechanism was identified in 43 patients, re-entry mechanism was identified in 22 patients. In 30 cases the AT was localized in the RA, in 28 cases in the LA, and in 7 patients we identified both RA and LA localization. In our control cohort, 39 patients were included with AT (31 de novo (79.5%), 6 post-PVI (15.4%), and 2 post-MAZE (5.1%) patients). Focal mechanism was identified in 20 patients and re-entry mechanism was identified in 19 patients. In 21 cases the AT was localized in the RA, in 14 cases in the LA, and in 4 patients we identified both RA and LA localization. Additional patient demographics and clinical data are summarized in Table 1 and illustrated in Fig. 2.

**Table 1:** Baseline patient demographics.

	AcQMap n = 147	CARTO n = 91	p-value
Age (years)	59.3 ± 12.1	59.4 ± 12.2	0.94
Female	50/136 (36.7%)	37/89 (41.5%)	0.47
Height (cm)	177.7 ± 10.2	176.3 ± 11.2	0.35
Weight (kg)	86.6 ± 15.7	85.6 ± 15.3	0.61
BMI	27.2 ± 3.9	27.2 ± 4.4	0.65
Heart failure	8 (5.4%)	1 (1.1%)	0.08
Ischemic heart disease	16 (10.9%)	3 (3.3%)	0.02
Hypertension	57 (38.7%)	39 (42.8%)	0.89
Cardiomyopathy	3 (2.0%)	8 (8.7%)	0.35
Diabetes	18 (12.2%)	11 (12.0%)	0.84
Dyslipidemia	16 (10.9%)	4 (4.4%)	0.06
CVA or TIA	16 (10.9%)	5 (5.5%)	0.16
OSAS	15 (10.2%)	3 (3.3%)	0.04
Antiarrhythmic medication	84 (57.1%)	61 (67.0%)	0.48
Preoperative LVEF	54.0 ± 6.3	54.6 ± 7.0	0.59
LA diameter (mm)	44.3 ± 7.4	43.9 ± 5.6	0.75
LA volume (mL)	80.9 ± 23.3	67.3 ± 13.5	0.09
LAVI (mL/m^2^)	38.9 ± 11.2	40.2 ± 16.3	0.63
TAPSE (mm)	20.5 ± 5.4	23.1 ± 4.7	0.01
Redo procedure	76 (51.7%)	17 (18.6%)	<0.01

Values are given as mean ± SD, n (%). BMI indicates body mass index; AAD, antiarrhythmic drug; LA, left atrium; LVEF, left ventricular ejection fraction; LAVI, left atrial volume index; IHD, Ischemic heart disease; CVA/TIA, cerebrovascular accident/transient ischemic attack; OSAS, obstructive sleep apnea syndrome; TAPSE, tricuspid annular plane systolic excursion.

### 3.2 Primary endpoint: Safety data

**Fig. 3:**
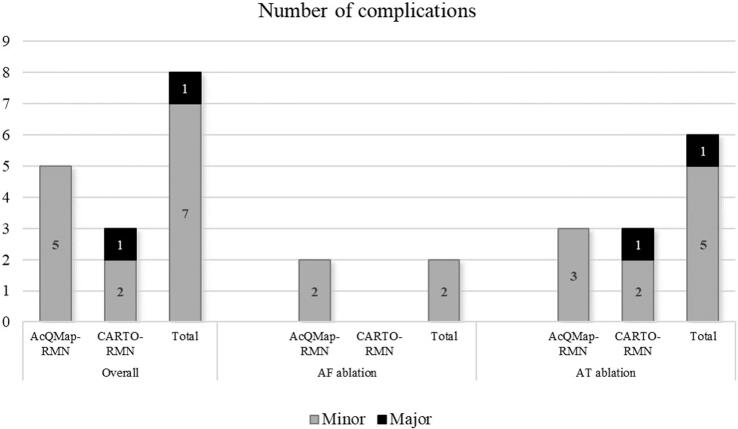
Procedure related complications. Minor and major complications are described in atrial fibrillation and atrial tachycardia ablation procedures guided by the AcQMap-RMN and CARTO-RMN systems separately (n).

There were no differences in safety between the AcQMap-RMN and CARTO-RMN patient groups (intra/post-procedural complications in 5 (3.4%) vs. 3 (3.3%) patients, p = 1.0) (see Fig. 3). There were no major intra- or post-procedural complications reported in the AcQMap-RMN patient group. Four patients were documented with groin hematoma, and one patient was documented with transient ischemic attack as a minor post-procedural complication (3.4%), which did not require a longer hospitalization time. In the CARTO-RMN group one patient was documented with phrenic nerve injury with long-standing diaphragm paralysis as a major complication. Another patient had groin hematoma, and one patient developed post-procedural palatal bleeding as minor complication.

### 3.3 Procedural and radiofrequency ablation data

In the overall group procedure durations were longer in all AcQMap-RMN guided ablations (172.5 ± 52.6 vs. 129.6 ± 47.7 min, p < 0.01), as well as fluoroscopy doses were higher (181.0 (IQR 110.5–320.0) vs. 132.0 (IQR 68.5–220.5) mGy, p = 0.02). Fluoroscopy doses were higher during AT ablations in the AcQMap-RMN group (152.0 vs. 74.9 mGy, p < 0.01). There were no differences in radiation doses during paroxysmal and persistent AF ablation between the AcQMap-RMN and CARTO-RMN groups (PAF 128.5 vs. 145.0 mGy, p = 0.91; persAF 256.0 vs. 187.5 mGy, p = 0.18). We found no differences in radiofrequency (RF) application number and application duration during paroxysmal AF and AT ablations between the treatment and control groups (PAF application number 21.0 vs. 19.0, p = 0.81; PAF application duration 1417.3 vs. 1616.6 s, p = 0.42; AT application number 24.0 vs. 18.0, p = 0.16; AT application duration 1249.3 vs. 1120.5 s, p = 0.51). Substrate ablation was performed in 45 patients (63.4%) in the AcQMap-RMN group, and in 6 patients (11.5%) in the CARTO-RMN group. We documented a higher number of RF applications in persistent AF ablation in the AcQMap-RMN group compared to the control group (37.0 vs 15.0, p < 0.01). Acute success was achieved in 137/147 patients (93.2%) in the AcQMap-RMN group, and 81/91 (89.0%) in the CARTO-RMN group (p = 0.43). Procedural data are summarized in Table 2.

**Table 2:** Procedural data.

	AcQMap-RMN	CARTO-RMN	p-value
	Paroxysmal AF ablation		
Procedure time (min)	158.9 ± 35.0	112.0 ± 39.5	< 0.01
Radiation dose (mGy)*	128.5 (100.2–206.2)	145.0 (89.5–231.0)	0.91
No of applications*	21.0 (13.1–37.2)	19.0 (8.5–37.5)	0.81
Application duration (s)	1417.3 ± 909.4	1616.6 ± 941.6	0.42
	Persistent AF ablation		
Procedure time (min)	176.5 ± 46.2	134.3 ± 51.9	<0.01
Radiation dose (mGy)*	256.0 (168.0–480.0)	187.5 (104.5–394.0)	0.18
No of applications*	37.0 (23.0–47.0)	15.0 (11.5–20.5)	<0.01
Application duration (s)	1919.3 ± 935.2	1887.4 ± 911.8	0.90
	AT ablation		
Procedure time (min)	174.6 ± 61.7	144.7 ± 49.1	0.01
Radiation dose (mGy)*	152.0 (84.2 – 299.5)	74.9 (27.5 – 169.7)	< 0.01
No of applications*	24.0 (10.7 – 48.2)	18.0 (8.2 – 38.0)	0.16
Application duration (s)	1249.3 ± 994.7	1120.5 ± 888.4	0.51

Values are given as mean ± SD, n (%), and * values are presented as median and interquartile range (IQR)

### 3.4 Learning curve

**Fig. 4:**
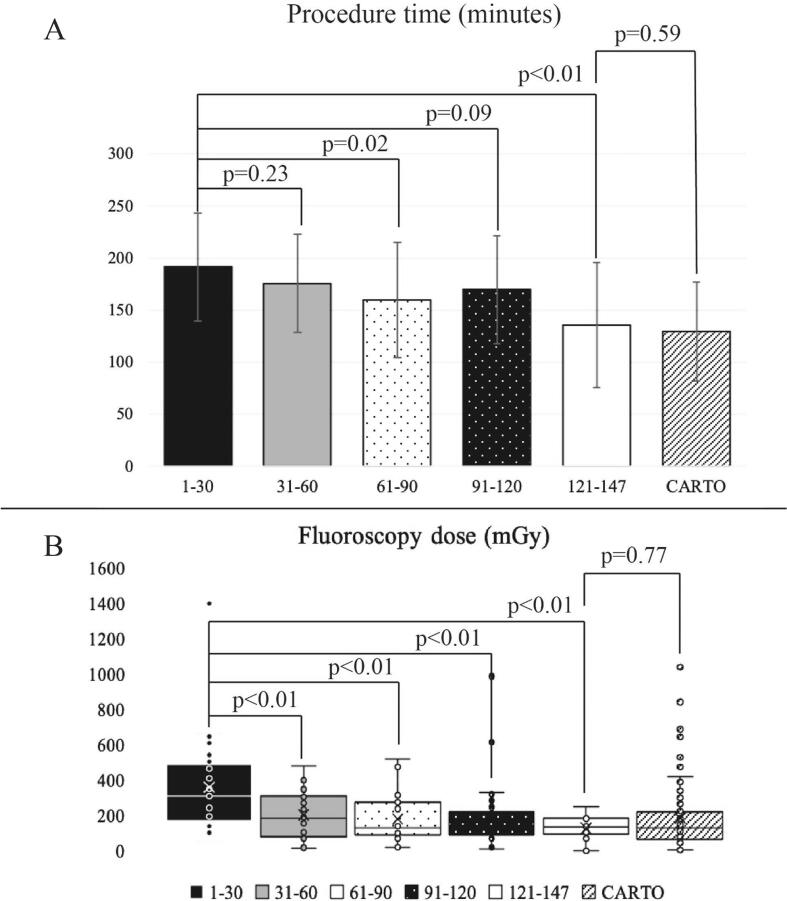
AcQMap-RMN learning curve. Patients were divided into groups of thirty. Group ‘1-30′ meaning the first 30 patients, and group ‘121-147′ meaning the most recent patients included in our study. We compared data from the most recently included ‘121-147′ patient group to the overall CARTO-RMN patients. Panel A illustrates the learning curve for AcQMap-RMN procedure duration. Panel B illustrates a significant decrease in fluoroscopy use over the study period.

Based on procedure time and fluoroscopy dose, a learning curve for AcQMap-RMN procedures was drawn (Fig. 4), which shows that late AcQMap-RMN procedures were significantly shorter and required lower fluoroscopy doses compared to early procedures (191.7 vs. 135.5 min, p < 0.01; 310.1 vs. 134.0 mGy, p < 0.01). Comparing the last 30 AcQMap-RMN procedures (19 AT, 6 PAF, 5 persAF) to the overall CARTO-RMN group we found no significant differences in procedure time and fluoroscopy dose (135.5 vs. 129.6 min, p = 0.59; 134.0 vs. 132.0 mGy, p = 0.77).

### 3.5 Follow-up data

The AcQMap-RMN was non-inferior to CARTO-RMN guided paroxysmal AF ablation regarding efficacy (1 year recurrence 6.6% vs. 12.5%, p = 0.58), and there were no significant differences in percentage of redo procedures between the two groups (0.0% vs. 3.0%, p = 1.00). The AcQMap-RMN system showed significant improvement in persistent AF ablation outcomes compared to the CARTO-RMN guided ablations (1 year recurrence 36.6% vs. 75.0%, p = 0.04 ;). Significant efficacy improvements were observed in the AcQMap-RMN group regarding the number of redo procedures following persistent AF ablations (12.7% vs. 38.8%, p = 0.03). AcQMap-RMN demonstrated significant improvement in de novo AT ablation outcomes (3-months recurrence 2.8% vs. 38.7%, p < 0.01; 1 year recurrence 17.1% vs. 38.7%, p = 0.05). There were no differences in percentage of redo procedures between the two groups (2.8% vs. 6.4%, p = 0.59). Follow-up data are summarized in Table 3.

**Table 3:** Follow-up data.

	AcQMap-RMN	CARTO-RMN	p-value
Paroxysmal AF follow-up data			
Recurrence 12 months	1/15 (6.6%)	1/8 (12.5%)	0.58
Persistent AF follow-up data			
Recurrence at 12 months	11/30 (36.6%)	9/12 (75%)	0.04
De novo AT follow-up data			
Recurrence at 3 months	1/35 (2.8%)	12/31 (38.7%)	< 0.01
Recurrence within 1 year	6/35 (17.1%)	12/31 (38.7%)	0.05
Post-PVI/post-MAZE AT follow-up data			
Recurrence at 3 months	7/22 (31.8%)	1/8 (12.5%)	0.39
Recurrence within 1 year	7/22 (31.8%)	3/8 (37.5%)	1.00

Values are given as n (%).

## 4 Discussion

The main finding of our report is that the AcQMap-RMN guided ablation is non-inferior to CARTO-RMN guided ablation in procedural safety. It also demonstrated to be non-inferior in the treatment of paroxysmal AF, and superior to CARTO-RMN integration in persistent AF and AT ablation.

### 4.1 Major advantages of RMN guided ablation: Safety and efficacy

To achieve a successful CA procedure, accurate substrate localization is a crucial requirement, followed by optimal radiofrequency energy delivery provided by good catheter-tissue contact. Safe manual catheter manipulation is often challenging, and manual catheters are frequently limited by their predefined curve, which requires additional manual skills to maneuver in some cases. When maneuvering the stiff manual ablation catheter frequent fluoroscopic visualization is required, therefore radiation exposure during manual CA procedures can be extensive. Furthermore, some anatomic regions are difficult to reach, and suboptimal catheter positioning may result in insufficient lesion formation [13]. The introduction of RMN technology in modern electrophysiology led to more effective and safe CA procedures [11], [13] for a wide spectrum of arrhythmias. The RMN system offers precise and stable catheter manipulation and eliminates acute complications during procedures almost entirely [5], [14]. In the last decades RMN has been associated with very few complications and gained a legacy within the EP community of being one of the safest platforms in the treatment of arrhythmias. Introducing a new integration possibility with the AcQMap system introduced larger amount of manual component into RMN procedures. Our current data supports the hypothesis that despite this the procedures have maintained an excellent safety profile. In general, RMN guided procedures have also been associated with significantly shorter fluoroscopy times as compared to the manual approach [6].

As earlier studies show, the RMN system has demonstrated superiority to conventional manual methods in ventricular tachycardia ablation [6], [15], [16], [17], and it has been proven feasible and non-inferior to manual methods in AF ablation [18], [19]. Our present study is the first that demonstrates improved outcome using the newly integrated mapping module.

### 4.2 RMN and mapping systems for AF and ATs

Our data suggests that integrating the AcQMap high-resolution mapping system in the RMN system offers new possibilities in CA procedures. Initially this came at the cost of longer procedures and increased fluoroscopy use. Possible reasons include that this technology is at its early stage, it can face technical issues during procedures and it has fewer automatic features. Our results show progressive reduction in procedure duration and fluoroscopy use, with reasonable procedure durations and fluoroscopy doses observed after passing a rather slow learning curve (30 procedures). We demonstrated comparable procedure times and fluoroscopy doses between the two systems (AcQMap-RMN and CARTO-RMN) as we advanced along the learning curve. Even though the AcQMap-RMN integration is safe and feasible, it increases fluoroscopy time in paroxysmal AF ablation, resulting in longer ablation procedures. Therefore, we do not specifically recommend the use of AcQMap-RMN integration over CARTO-RMN systems in paroxysmal AF ablation. Similarly to the results of the UNCOVER AF trial, AcQMap-RMN integration demonstrates high long-term success rates in CA for non-PV triggers in addition to PVI for persistent AF [20]. Our results show improvements in outcome using the AcQMap-RMN integration compared to the CARTO-RMN systems. We believe this may be a step forward in persistent AF ablation.

Previous studies show that using the RMN system in AT ablation reduces fluoroscopy time compared to manual methods, however acute and long-term success rates demonstrated no significant improvements with the RMN system [21], [22]. With AcQMap-RMN integration we documented good outcome improvements for de novo AT patients. Our results suggest that AcQMap-RMN integration decreases 1-year recurrence rates from 38% (with CARTO-RMN) to 17%. Furthermore, due to the single-beat feature of the AcQMap mapping system, a previously rejected patient population with highly symptomatic short runs of AT can be treated with high long-term success rates [23].

This is the first comparative study reporting on the AcQMap-RMN and CARTO-RMN integration in PAF, persAF and AT ablation. Further studies with targeted focus on each atrial arrhythmia separately are therefore suggested.

### 4.3 Limitations

The AcQMap technology requires fluoroscopic confirmation of basket catheter position, therefore fluoroscopy doses are relatively high compared to the CARTO-RMN group. Primarily, this study was powered to asess procedural safety, therefore, subgroups such as AT patients were not undertaken to power calculation. For a more in depth comparison of CA procedures for patients with AT, we recommend further studies focusing on the above mentioned subgroup only. Due to the nature of AcQMap mapping technology, the planning strategy in our institution allows the majority of previously unsuccessfully ablated patients to be planned for a redo procedure with this system. This may be the reason why there are more redo procedures in the AcQMap-RMN group than in the CARTO-RMN patient group. Seemingly, this difference did not affect outcome parameters. The difference in the use of a substrate ablation between the two groups is due to the fact that the CARTO system does not offer a commercially available specific feature for substrate mapping. The AcQMap mapping was specifically designed for substrate mapping, and using the CARTO system empiric substrate ablation can be performed.

## 5 Conclusions

AcQMap-RMN integration has no negative impact on the excellent safety profile of the RMN guided ablations, and is non-inferior to CARTO-RMN integration. It also demonstrated to be non-inferior in the treatment of paroxysmal AF, and superior to CARTO-RMN integration in persistent AF and AT ablation. However, in order to achieve such improvements it requires longer procedure times and uses more fluoroscopy during the early learning phase. The AcQMap-RMN integration allows for a safe and personalized treatment option for patients suffering from a vast spectrum of arrhythmias.

## Funding sources

This research did not receive any specific grant from funding agencies in the public, commercial, or not-for-profit sectors.

## Declaration of Competing Interest

The authors declare that they have no known competing financial interests or personal relationships that could have appeared to influence the work reported in this paper.
